# Correction to: Identification of pathways modulating vemurafenib resistance in melanoma cells via a genome-wide CRISPR/Cas9 screen

**DOI:** 10.1093/g3journal/jkac024

**Published:** 2022-02-16

**Authors:** Corinna Jie Hui Goh, Jin Huei Wong, Chadi El Farran, Ban Xiong Tan, Cynthia R Coffill, Yuin-Han Loh, David Lane, Prakash Arumugam

*G3: Genes|Genomes|Genetics*, 2021, https://doi.org/10.1093/g3journal/jkab069

In the originally published version of this manuscript, Yuin-Han Loh’s name was spelled incorrectly. This has been corrected.

